# Evaluation of the aorta in infants with simple or complex coarctation of the aorta using CT angiography

**DOI:** 10.3389/fcvm.2022.1034334

**Published:** 2023-01-09

**Authors:** Hui-Jun Xiao, A-Lai Zhan, Qing-Wen Huang, Rui-Gang Huang, Wei-Hua Lin

**Affiliations:** Department of Radiology, Zhangzhou Affiliated Hospital of Fujian Medical University, Zhangzhou, China

**Keywords:** CTA, CoA, diagnosis, aortic dilatation, infant

## Abstract

**Objective:**

To assess aortic dilatation and determine its related factors in infants with coarctation of the aorta (CoA) by using computed tomography angiography (CTA).

**Methods:**

The clinical data of 55 infantile patients with CoA diagnosed by CTA were analyzed retrospectively. Aortic diameters were measured at six different levels and standardized as Z scores based on the square root of body surface area. The results of simple and complex CoA were compared. Univariate and multivariate logistic regression were used to analyze the effects of sex, age, hypertension, degree of coarctation, CoA type, bicuspid aortic valve (BAV), and other factors related to aortic dilatation.

**Results:**

In total, 52 infant patients with CoA were analyzed, including 22 cases of simple CoA and 30 cases of complex CoA. The ascending aorta of the infants in the simple CoA group and the complex CoA group were dilated to different degrees, but the difference was not statistically significant (50.00% vs. 73.33%, *P* = 0.084, and 2.05 ± 0.40 vs. 2.22 ± 0.43 *P* = 0.143). The infants in the complex CoA group had more aortic arch hypoplasia than those in the simple CoA group (33.33% vs. 9.09%, *P* = 0.042). Compared to the ventricular septal defect (VSD) group, the Z score of the ascending aorta in the CoA group was significantly higher than that in the VSD group (*P* = 0.023 and *P* = 0.000). A logistic retrospective analysis found that an increased degree of coarctation (CDR value) was an independent predictor of ascending aortic dilatation (adjusted OR = 0.002; *P* = 0.034).

**Conclusion:**

Infants with simple or complex CoA are at risk of ascending aortic dilatation, and the factors of ascending aortic dilatation depend on the degree of coarctation. The risk of aortic dilatation in infants with CoA can be identified by CTA.

## Introduction

Coarctation of the aorta (CoA) accounts for 5 to 9% of all congenital heart malformations (1, 2). As the course of the disease progresses, the patient may develop hypertension, left heart failure, and aortic dilatation, and CoA may even cause aortic aneurysm, aortic dissection (3, 4). In recent years, the perioperative morbidity and mortality of CoA in children and adults have been greatly reduced (5). However, recent follow-up studies have shown that after surgical correction of CoA, patients were still at risk of developing developmental restenosis, hypertension, and aortic aneurysms (6–8). In fact, the early state of an aortic aneurysm is dilation of the aorta. Studies showed that not only untreated patients with CoA were prone to worsen aortic dilatation, but also patients who received treatment still had the risk of aortic dilatation (9, 10). Meanwhile, infants with a degree of root dilation may maintain the dimensions over the course of many years, and conversely infants with normal roots (and a BAV) may develop root dilation as young adults. Dynamic follow-up of infants with CoA can provide early warning information. Therefore, it is necessary to evaluate the inner diameter of the aorta in infants with CoA and to explore the relevant factors affecting aortic dilatation. At present, multi-slice spiral computed tomography angiography (CTA) has been widely used to evaluate congenital heart disease (11, 12). Some studies have shown that patients with complex CoA have a more severe developmental spectrum than patients with simple CoA (13). We conducted a retrospective study to evaluate and quantify the dilatation of the entire thoracic aorta in infants with CoA. In addition, we compared the results of complex and simple CoA to identify determinants of aortic dilatation.

## Materials and methods

According to the inclusion and exclusion criteria, 55 infants with CoA were selected as the research subjects from January 2020 to August 2022. All infants underwent routine CTA and transthoracic echocardiography before surgery. The baseline data, echocardiography, CTA, and other examinations of the patients were retrospectively collected and obtained from medical records. The inclusion criterion was as follows: patients diagnosed with CoA by CTA and echocardiography before surgery. The exclusion criteria were as follows: (1) insufficient clinical data; (2) associated with deformities, such as double aortic arches, right ventricular double outlet, tetralogy of Fallot, and supra-aortic stenosis; (3) combined with Turner syndrome, Marfan syndrome, Loeys–Dietz syndrome, mucopolysaccharidosis, and other diseases; and (4) combined with aortic compression.

The present study included two groups of CoA. The first group was the simple CoA group, including infantile patients with or without patent ductus arteriosus (PDA). This group also included infantile patients with small atrial septal defect (ASD) or patent foramen ovale (PFO) who did not need surgical treatment. The second group was the complex CoA group, including infantile patients with ventricular septal defect (VSD) and/or ASD/PFO, and some of them also had PDA. For additional comparisons, we set up a third group as a simple VSD group.

Aortic arch hypoplasia was defined as a diameter of the proximal or distal transverse arch less than 50% of the diameter of the ascending aorta (14). The diagnostic criteria of hypertension included children less than 1 year old who were diagnosed according to the summary table of neonatal BP values compiled by Dionne et al. (15). The anatomical structure of aortic arch could be directly displayed by three-dimensional reconstruction technique, which could effectively guide the operation. Therefore, CTA was used as a routine examination for infants with CoA. This study was approved by our institution’s research ethics committee.

### CT angiography

A GE Revolution 256-slice multislice CT scanner equipped with a 16 cm wide detector was used in this study, and the rotation of the tube was 0.28 s. The time resolution of cardiovascular CT imaging can be improved by combining with snapshot freeze (SSF) algorithm reconstruction technology, a new motion correction algorithm that was developed to compensate for coronary artery motion and optimize diagnostic utility in CTA images (16). Under the condition of any heart rate or arrhythmia, the whole heart scan can be completed by an axial scan in one cardiac cycle, which greatly reduces the cardiac and respiratory movement artifacts and improves the diagnostic efficiency and image quality of the disease (17). At the same time, ASiR-V reconstruction technology has the advantages of real-time reconstruction, which further reduces noise and improves low density (18).

All infants were given oral 10% chloral hydrate sedation (dose 0.5 ml/kg) before the examination. The scanning range was from the entrance of the chest to 5 cm below the left diaphragm. The following scanning parameters were used: ECG-controlled prospective axial scanning; 2.5 mm slice spacing; 2.5 mm slice thickness; 0.625 mm reconstructed slice spacing; tube voltage of 70–90 kV, tube current of 100–200 mAs. For the contrast agent, lopromide injection (300 mg/ml iodine) was administered by a high-pressure syringe through peripheral veins, such as the hand and dorsum of the foot, at a dose of 1.5–2 ml/kg. For image post-processing and analysis, the original data were automatically reconstructed by the machine, and the reconstructed thin-layer image was transmitted to the image post-processing workstation for image reconstruction. Methods, including maximum intensity projection (MIP), multiplanar reconstruction (MPR), and volume rendering (VR), were used to visualize intracardiac structures, cardiac great vessel connections, extracardiac great vessels, and collateral vessels. The average effective radiation dose of CTA for all infants was 0.3 ± 0.05 mSv. All the images in this study met the needs of clinical diagnosis.

### Image analysis

**FIGURE 1:**
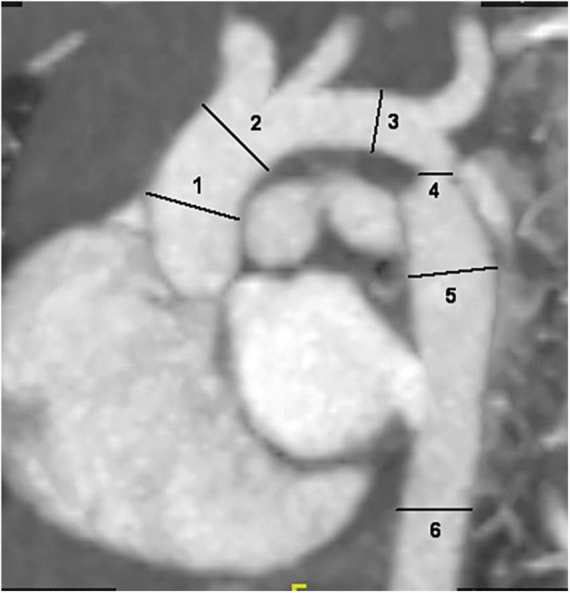
Sagittal multiplanar reformatted image shows the measurement of aortic diameters at different levels: 1. ascending aorta; 2. pre-coarctation aorta; 3. aortic arch; 4. site of coarctation; 5. descending aorta after the site of coarctation; 6. descending aorta at level of diaphragm.

The internal diameter of the aorta at all levels and concomitant cardiac malformations, including bivalvular aortic valve (BAV), aortic arch hypoplasia, PDA, VSD and ASD, were recorded. Two radiologists performed image measurements at the same time, and the average of both measurements was calculated to reduce the error of the data. The intracardiac malformations and cardiac valves were evaluated by transthoracic echocardiography. The diameter of the aorta was measured at the following six locations: ascending aorta at its maximum diameter (ascending aorta); aorta proximal to the origin of the brachiocephalic trunk (pre-coarctation aorta); aortic arch at the largest size (aortic arch); coarctation site at the narrowest size (coarctation site); widest region of the descending aorta (post-coarctation aorta); and descending aorta at the level of the diaphragm (Figure 1; 19–21). The aorta measurement was performed along the vertical direction of the long axis of the aorta in the median sagittal plane of the aortic arch. The ratio of the aortic diameter at the coarctation site to that at the diaphragm (coarctation site-diaphragm ratio, CDR) was calculated to describe the degree of coarctation as follows: CDR < 75%, diagnosed as aortic coarctation; 50% < CDR > 75%, diagnosed as mild coarctation; and CDR < 50%, diagnosed as severe coarctation (22, 23). To account for growth-related changes, the aortic diameter was normalized to a Z score, which was the ratio of aortic diameter to the square root of body surface area. If the Z score was more than 2, aortic dilatation was considered (24, 25). The body surface area (BSA) was calculated by the DuBois formula (26) and aortic dilatation was defined as a main artery Z score > 2.0 (24).

### Statistical analysis

Statistical analysis was performed using SPSS 23.0 software. All measurement data were tested for normality. A *t*-test was used if the data conformed to the normal distribution, and non-parametric tests were used if the data did not conform to the normal distribution. Univariate analysis was used to evaluate the possible related factors with the occurrence of ascending aortic dilatation, and multivariate binary logistic regression analysis was used for factors with *p* < 0.15 in univariate analysis. One-way analysis of variance (ANOVA) was used to compare the Z scores among the three groups, and the results were plotted in the form of histograms by GraphPad Prism (8.0) software. *P* < 0.05 was considered statistically significant.

## Results

Three of the infants with CoA were excluded from the study because they had a double outlet of the right ventricle, double aortic arch, and tetralogy of Fallot. In total, 52 cases with CoA were selected for further analysis, including 22 cases in the simple CoA group and 30 cases in the complex CoA group. In addition, we included 25 cases with simple VSD in the VSD group (no combined with CoA). The basic characteristics of the three groups are shown in Table 1. There were 13 cases of mild CoA and 9 cases of severe CoA in the simple CoA group, and there were 10 cases of mild CoA and 20 cases of severe CoA in the complex CoA group; there was no significant difference in the number of cases with mild and severe CoA between the two CoA groups (*p* = 0.065). There were four cases of BAV in the simple CoA group and eight cases of BAV in the complex CoA group, but there was no significant difference in the number of cases with BAV between the two groups (*P* = 0.447). In addition, there were eight cases of hypertension in the simple CoA group and 17 cases of hypertension in the complex CoA group, but the difference in the number of hypertension cases between the two groups was not statistically significant (*P* = 0.148) (Table 1). There was one case complicated with severe cardiac insufficiency did not show hypertension in the simple CoA group. Most severe CoA cases were associated with varying degree of cardiac insufficiency in both groups.

**TABLE 1:** General characteristics of the population.

Characteristics	Simple (*n* = 22)	Complex (*n* = 30)	VSD (*n* = 25)	*P*
Age (month)	2.88 ± 1.01	2.43 ± 1.28	2.63 ± 1.01	0.368
Male (%)	13 (59.10)	15 (50.00)	15 (60.00)	0.710
BSA (m^2^)	0.28 ± 0.11	0.25 ± 0.08	0.27 ± 0.10	0.454
Severe CoA (%)	9 (40.91)	20 (66.67)	–	0.065
Hypertension (%)	8 (36.36)	17 (56.67)	–	0.148
**Associated cardiovascular malformations**				
BAV (%)	4 (18.18)	8 (26.67)	–	0.477
PDA (%)	10 (45.45)	12 (40.00)	–	0.694
VSD (%)	0 (0)	25 (83.33)	–	–
ASD/PFO (%)	7 (31.81)	15 (50.00)	–	0.190

Data relate to the number of patients (percentage), or mean ± standard values.

Severe CoA, severe coarctation of aorta; BAV, bicuspid aortic valve; PDA, patent ductus arteriosus; VSD, ventricular septal defect; ASD, atrial septal defect; PFO, patent foramen ovale.

The CT measurement results showed that among the 52 infantile patients, the ascending aorta, descending aorta, pre-coarctation, and aortic arch were dilated in 33, 21, 17, and 6 cases, respectively. In the simple CoA group, 11 patients had dilated ascending aortas, and 7 patients had dilated descending aortas. In the complex CoA group, 22 patients had dilated ascending aortas, and 14 patients had dilated descending aortas. In both CoA groups, the main area of dilation occurred in the ascending aorta. The number of ascending dilatations in the complex CoA group was slightly higher than that in the simple CoA group, but the difference was not statistically significant (*P* = 0.084). Further comparison of the Z score showed that the Z scores of the ascending aorta and descending aorta in the complex CoA group were 2.22 ± 0.43 and 2.00 ± 0.34, respectively, which were slightly higher than those in the simple CoA group (2.05 ± 0.40 and 1.91 ± 0.29, respectively), but the difference was not statistically significant (*P* = 0.143 and *P* = 0.356, respectively). There were two cases of aortic arch hypoplasia in the simple CoA group and 10 cases of aortic arch hypoplasia in the complex CoA group, and the difference in the number of aortic arch hypoplasia cases between the groups was statistically significant (*P* = 0.042) (Table 2).

**TABLE 2:** Comparison of aortic dilatation and arch hypoplasia between the two groups.

	Simple (*n* = 22)	Complex (*n* = 30)	*P*
**Z score of aorta**			
Ascending aorta	2.05 ± 0.40	2.22 ± 0.43	0.143
Descending aorta	1.91 ± 0.29	2.00 ± 0.34	0.356
Pre-coarctation	1.85 ± 0.34	1.92 ± 0.32	0.468
Aortic arch	1.72 ± 0.22	1.75 ± 0.24	0.651
**Aortic dilation (%)**			
Ascending aorta	11 (50.00)	22 (73.33)	0.084
Descending aorta	7 (31.82)	14 (46.67)	0.281
Pre-coarctation	6 (27.27)	11 (36.67)	0.476
Aortic arch	2 (9.09)	4 (13.33)	0.639
Hypoplastic arch	2 (9.09)	10 (33.33)	0.042

Values are showed as mean ± SD or count (percent). Hypoplastic arch: Aortic arch hypoplasia. The aorta measurement was measured along the vertical direction of the long axis of the aorta in the median sagittal plane of the aortic arch.

**FIGURE 2:**
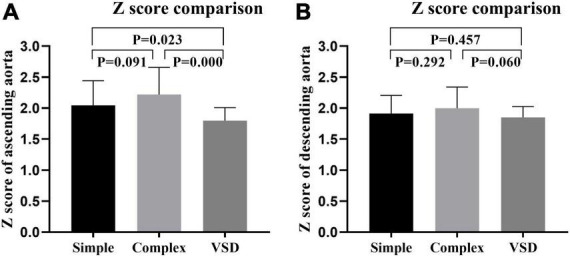
Z scores of the ascending **(A)** descending aorta **(B)** in the simple coarctation of the aorta (CoA) group, the complex CoA group, and the ventricular septal defect (VSD) group were compared.

To further verify the dilatation of the ascending aorta in infantile patients with CoA, we compared both CoA groups to the VSD group. The Z score of the ascending aorta in both CoA groups was significantly higher than that in the VSD group (*P* = 0.023 and *P* = 0.000, respectively). Although some infantile patients had descending aorta dilatation, there was no significant difference in the Z score of the descending aorta between the two CoA groups and the VSD group (*P* > 0.05). These results suggested that infantile patients with CoA are mainly characterized by ascending aortic dilatation (Figure 2).

We divided infants with CoA into groups with or without ascending aortic dilatation and compared the related factors between the two groups by *t*-test or chi-square test. Comparison of the related factors indicated that there was no significant difference in BAV, aortic arch hypoplasia, complexity, or hypertension between the ascending aortic dilatation group and the non-ascending aortic dilatation group; however, there was a significant difference in the degree of aortic coarctation between the two groups (*P* = 0.004) (Table 3). Univariate analysis was used to determine the factors related to ascending aortic dilatation, and the results showed that CoA complexity, CDR, and BAV were related to aortic dilatation (*p* < 0.15). To reduce the collinearity in multivariate analysis, multivariable binary logic regression analysis was used to correct confounding factors, which indicated that only the degree of coarctation (CDR value) was independently related to aortic dilatation (adjusted OR = 0.002; 95% CI 0.00–0.62; *P* = 0.034) (Table 4).

**TABLE 3:** Analysis of factors affecting dilatation of ascending aorta.

	Dilatation (*n* = 33)	Non-dilatation (*n* = 19)	*P*
BAV (%)	10 (30.30)	2 (10.53)	0.142
Hypoplastic Arch (%)	9 (27.27)	3 (15.79)	0.349
Complex CoA (%)	21 (63.64)	9 (47.37)	0.253
Hypertension (%)	17 (51.52)	8 (42.11)	0.513
Severe CoA (%)	22 (66.67)	7 (36.84)	0.037
CDR	0.51 ± 0.13	0.41 ± 0.11	0.004

CDR, coarctation site–diaphragm ratio.

**TABLE 4:** Univariate and multivariate analyses for the presence of the ascending aortic dilation.

	Univariate		Multivariate	
Variable	Unadjusted OR (95% CI)	*P*	Adjusted OR (95% CI)	*P*
Gender	1.508 (0.49–4.69)	0.478	–	–
Age	1.296 (0.72–2.33)	0.386	–	–
Hypertension	1.461 (0.47–4.56)	0.514	–	–
CoA complexity	2.750 (0.86–8.80)	0.088	1.933 (0.53–7.11)	0.321
Severity of CoA (CDR)	0.001 (0.00–0.16)	0.008	0.002 (0.00–0.62)	0.034
BAV	3.696 (0.72–19.10)	0.119	1.186 (0.17–8.20)	0.863

## Discussion

Coarctation of the aorta occurs as a simply isolated disorder or in a more complex form, in which it is combined with associated other congenital intracardiac abnormalities (1, 2). The presence of these abnormalities in patients with complex CoA suggests that these patients have a more severe developmental spectrum. Due to the high late complication rate, some studies have suggested that CoA, rather than being an isolated entity characterized by a simple mechanical obstruction of the aorta, are a general disease of the cardiovascular system (26). In the present study, complex CoA was often associated with hypoplasia of the aortic arch; that is, hypoplasia of the aortic arch was more common in cases of CoA with intracardiac malformation. This concept was supported by the hemodynamic underdeveloped theory of the pathogenesis of CoA (27). We also made a comparison between the infantile patients with simple and complex coarctation of the aorta and the simple VSD group and found that the Z score of the ascending aorta in the complex and simple groups was significantly different from that in the VSD group, indicating that the ascending aorta of infantile patients with CoA may be dilated to different degrees. At the same time, we conducted multivariate analysis of the Z score of the ascending aorta in infantile patients with CoA and found that complexity was not a risk factor. Thus, these findings suggested that the hemodynamic effect of CoA mainly lies in the degree of coarctation, while intracardiac deformities, such as VSD, ASD, and PDA, do not increase aortic wall shear stress. Different types of VSD might have different hemodynamic effects, but the type of VSD in the complex CoA group were all the peri-membranous VSD.

Compared to the VSD group, there was no obvious dilatation of the aorta in infants with CoA, except for the ascending aorta. A previous study has suggested that the changes in the functional and structural characteristics of the aorta are limited to the narrowing of the anterior artery, while the morphology of the posterior artery is retained (28) which was consistent with the present results. However, Zhang et al. showed that with the increase in the degree of coarctation, the number of high-intensity vortices significantly increase, leading to dilatation of the lower aorta (9). The non-dilatation of the lower aorta (descending aorta) may have been related to the following factors: (1) the selected infantile patients were relatively young, and the action time on the wall of the tube was short; and (2) the formation of collateral circulation in CoA alleviates the blood flow in the lower segment, thus weakening the impact on the wall of the lower segment. Previous studies have reported that approximately half of CoA patients are associated with BAV (29). In children with BAV, NadorlikH et al. suggested that abnormal genetics and hemodynamics may lead to remodeling of pathogenic cells and extracellular matrix in the proximal aorta, eventually leading to ascending aorta dilatation (30). In addition, some genes are related to the molecular basis of BAV, such as mutations in NOTCH1 and GATA5, which are associated with human mitral and aortic valves (30). Some scholars have suggested that genetic abnormalities that lead to BAV may also lead to histopathological changes, which are prone to aortic aneurysms (30). In the present study, the percentage of patients with BAV was only 23.1%. Considering the small sample size and the small number of positive cases with BAV, the conclusion was quite different.

Further analysis of the risk factors for the ascending aortic dilatation found that hypertension, gender, age, and the complexity of CoA and BAV were not risk factors for the ascending aortic dilatation. Only the degree of coarctation was an independent risk factor for the ascending aortic dilatation. Similarly, Sehested et al. histologically analyzed arterial tissue in patients with CoA and reported that the arrangement of elastin in the middle layer of the ascending aorta is disrupted compared to the posterior segment of aortic coarctation, and they also reported that the content of collagen is increased and that the mass of smooth muscle is decreased in the middle layer of the ascending aorta (31). The presence of CoA disrupts the blood flow of the ascending aorta and harms the hemodynamics of the aorta, leading to dilatation of the ascending aorta (32). These hemodynamics have been demonstrated to aggravate vascular endothelial dysfunction, arterial smooth muscle dedifferentiation, and endothelial thickening, while abnormal aortic wall or blood flow changes may lead to abnormal aortic walls and are prone to complications, such as aortic dilatation, aortic aneurysm, and endovascular inflammation (28, 33). Zhao et al. (24) showed ascending aorta dilatation and suggested that the degree of dilatation is related to the degree of coarctation, which agreed with the present study; however, their results also showed that the descending aorta was accompanied by dilatation, which was quite different from our findings. This difference may be due to the different ages of the subjects. In the present study, 48.08% of the infantile patients with CoA were complicated with hypertension. Related studies have also shown that hypertension in CoA patients may lead to vascular dysfunction due to endothelial shear stress. In addition, hypertension may activate gene expression and lead to proliferation and hypertrophy of vascular smooth muscle (33). However, the present results suggested that there was no significant association between hypertension and ascending aortic dilatation, considering that they were both secondary manifestations of CoA. In the present study, 23.1% of the infantile patients were complicated with BAV. It is well known that the presence of BAV may lead to eccentric blood flow in the ascending aorta and significantly increase the shear stress of the aortic wall, resulting in dilatation of the ascending aorta (34). Sinning et al. showed that approximately 70% of children with CoA have BAV and that aortic dilatation is more common in children with CoA-BAV than in children with simple CoA (35). However, in the present study, CoA combined with BAV did not significantly affect ascending aorta dilatation, and we determined that BAV cannot be used as a predictor of ascending aortic dilatation, which contradicted previous studies (34). The differences may be due to several factors. First, the sample size was small, indicating that the incidence of BAV and the factors of aortic dilatation need to be further verified. Second, the subjects in the present study were young, and Sinning et al. reported that aortic dilatation in children with CoA-BAV progresses with age (35). Thus, it is necessary to conduct longitudinal studies in the future to confirm this conclusion. Finally, we considered that the pathophysiology of CoA-BAV was significantly different from that of isolated BAV disease in the present study.

Multi-slice spiral CT angiography has the following advantages: fast scanning speed, high spatial resolution, not affected by heart rate, and low radiation dose. The combination of original axial images and three-dimensional images has unique advantages in the diagnosis of CoA, which is incomparable to DSA and MRA, especially in the diagnosis of macrovascular malformations (17, 18). Rupture and dissection of the aorta, which develop from aortic dilatation, are considered to be important causes of death in patients with CoA (7). The present results showed that infantile patients with CoA may present with ascending aorta dilatation, and related studies have shown that with increasing age, the ascending aorta progressively dilates and increases the risk of aortic rupture (12). Therefore, for infantile patients with CoA, CTA scans should be routinely performed both pre-operatively and post-operatively to predict the possible risk of aortic dilatation.

The present study had several limitations. First, this study used data from a single institution, and it was a retrospective study with a small sample size, indicating that further verification with a large sample is required in the future. Fewer cases of BAV were included in this study, which might be related to the small sample size and might affect the statistical results. Second, we only performed a pre-operative evaluation (horizontal study). Because related studies have reported that the aorta of children with CoA dilate progressively with age, it is necessary to perform long-term follow-up (longitudinal study) after surgery in the future. In addition, studies should be performed in children of different ages to determine whether the aorta dilates progressively with age in children with CoA.

## Conclusion

Infants with simple and complex CoA are at risk of ascending aortic dilatation, and complex CoA is often associated with aortic arch hypoplasia. The degree of aortic dilatation in infants with CoA is mainly related to the degree of coarctation, and the condition of the aorta in infants with CoA can be evaluated by CTA.

## Data availability statement

The raw data supporting the conclusions of this article will be made available by the authors, without undue reservation.

## Ethics statement

The studies involving human participants were reviewed and approved by the Ethics Committee of Zhangzhou Affiliated Hospital of Fujian Medical University. Written informed consent to participate in this study was provided by the participants’ legal guardian/next of kin.

## Author contributions

H-JX designed the study, drafted the manuscript, and submitted the manuscript. A-LZ, Q-WH, R-GH, and W-HL collected and analyzed the data. All authors read the final version of this article and approved for publication.

## References

[1] Campbell M. Natural history of coarctation of the aorta. *Br Heart J.* (1970) 32:633–40. 10.1136/hrt.32.5.633 5470045 PMC487385

[2] Tronc F Curtil A Robin J Ninet J Champsaur G. Coarctation et son traitement chirurgical [Coarctation of the aorta and its surgical treatment]. *Arch Mal Coeur Vaiss.* (1997) 90(Suppl. 12):1729–36. 10.1016/S1010-7940(97)00114-0 9587458

[3] De Mey S Segers P Coomans I Verhaaren H Verdonck P. Limitations of doppler echocardiography for the post-operative evaluation of aortic coarctation. *J Biomech.* (2001) 34:951–60. 10.1016/S0021-9290(01)00043-4 11410178

[4] Basso C Boschello M Perrone C Mecenero A Cera A Bicego D An echocardiographic survey of primary school children for bicuspid aortic valve. *Am J Cardiol.* (2004) 93:661–3. 10.1016/j.amjcard.2003.11.031 14996606

[5] Zoghbi J Serraf A Mohammadi S Belli E Lacour Gayet F Aupecle B Is surgical intervention still indicated in recurrent aortic arch obstruction? *J Thorac Cardiovasc Surg.* (2004) 127:203–12. 10.1016/S0022-5223(03)01290-X 14752432

[6] Rakhra S Lee M Iyengar A Wheaton G Grigg L Konstantinov I Poor outcomes after surgery for coarctation repair with hypoplastic arch warrants more extensive initial surgery and close long-term follow-up. *Interact Cardiovasc Thorac Surg.* (2013) 16:31–6. 10.1093/icvts/ivs301 23059853 PMC3523616

[7] von Kodolitsch Y Aydin M Koschyk D Loose R Schalwat I Karck M Predictors of aneurysmal formation after surgical correction of aortic coarctation. *J Am Coll Cardiol.* (2002) 39:617–24. 10.1016/S0735-1097(01)01784-3 11849860

[8] Isner J Donaldson R Fulton D Bhan I Payne D Cleveland R. Cystic medial necrosis in coarctation of the aorta: a potential factor contributing to adverse consequences observed after percutaneous balloon angioplasty of coarctation sites. *Circulation.* (1987) 75:689–95. 10.1161/01.CIR.75.4.689 2951035

[9] Zhang X Luo M Fang K Li J Peng Y Zheng L Analysis of the formation mechanism and occurrence possibility of post-stenotic dilatation of the aorta by CFD approach. *Comput Methods Programs Biomed.* (2020) 194:105522. 10.1016/j.cmpb.2020.105522 32422474

[10] Suárez de Lezo J Romero M Pan M Suárez de Lezo J Segura J Ojeda S Stent repair for complex coarctation of aorta. *JACC Cardiovasc Interv.* (2015) 8:1368–79. 10.1016/j.jcin.2015.05.018 26315741

[11] Bromberg B Beekman R Rocchini A Snider A Bank E Heidelberger K Aortic aneurysm after patch aortoplasty repair of coarctation: a prospective analysis of prevalence, screening tests and risks. *J Am Coll Cardiol.* (1989) 14:734–41. 10.1016/0735-1097(89)90119-8 2768722

[12] Luijendijk P Franken R Vriend J Zwinderman A Vliegen H Winter M Increased risk for ascending aortic dilatation in patients with complex compared to simple aortic coarctation. *Int J Cardiol.* (2013) 167:827–32. 10.1016/j.ijcard.2012.02.014 22370370

[13] Gutgesell H Barton D Elgin K. Coarctation of the aorta in the neonate: associated conditions, management, and early outcome. *Am J Cardiol.* (2001) 88:457–9. 10.1016/S0002-9149(01)01704-0 11545779

[14] Backer C Mavroudis C. Congenital heart surgery nomenclature and database project: patent ductus arteriosus, coarctation of the aorta, interrupted aortic arch. *Ann Thorac Surg.* (2000) 69(Suppl. 4):S298–307. 10.1016/S0003-4975(99)01280-1 10798436

[15] Dionne J Abitbol C Flynn J. Hypertension in infancy: diagnosis, management and outcome. *Pediatr Nephrol.* (2012) 27:17–32. 10.1007/s00467-010-1755-z 21258818

[16] Sheta H Egstrup K Husic M Heinsen L Nieman K Lambrechtsen J. Impact of a motion correction algorithm on image quality in patients undergoing CT angiography: a randomized controlled trial. *Clin Imaging.* (2017) 42:1–6. 10.1016/j.clinimag.2016.11.002 27838576

[17] Pontone G Baggiano A Andreini D Guaricci A Guglielmo M Muscogiuri G Dynamic stress computed tomography perfusion with a whole-heart coverage scanner in addition to coronary computed tomography angiography and fractional flow reserve computed tomography derived. *JACC Cardiovasc Imaging.* (2019) 12:2460–71. 10.1016/j.jcmg.2019.02.015 31005531

[18] Papadakis A Damilakis J. Technical note: quality assessment of virtual monochromatic spectral images on a dual energy CT scanner. *Phys Med.* (2021) 82:114–21. 10.1016/j.ejmp.2021.01.079 33610006

[19] Becker C Soppa C Fink U Haubner M Müller-Lisse U Englmeier K Spiral CT angiography and 3D reconstruction in patients with aortic coarctation. *Eur Radiol.* (1997) 7:1473–7. 10.1007/s003300050319 9369517

[20] Hager A Kaemmerer H Rapp-Bernhardt U Blücher S Rapp K Bernhardt T Diameters of the thoracic aorta throughout life as measured with helical computed tomography. *J Thorac Cardiovasc Surg.* (2002) 123:1060–6. 10.1067/mtc.2002.122310 12063451

[21] Robicsek F. Post-stenotic dilatation of the great vessels. *Acta Med Scand.* (1955) 151:481–5. 10.1111/j.0954-6820.1955.tb10316.x 14398135

[22] Türkvatan A Akdur P Olçer T Cumhur T. Coarctation of the aorta in adults: preoperative evaluation with multidetector CT angiography. *Diagn Interv Radiol.* (2009) 15:269–74. 10.4261/1305-3825.DIR.2434-08.1 19847770

[23] Yu Y Wang Y Yang M Huang M Li J Jia Q Evaluating the severity of aortic coarctation in infants using anatomic features measured on CTA. *Eur Radiol.* (2021) 31:1216–26. 10.1007/s00330-020-07238-1 32885294

[24] Zhao Q Shi K Yang Z Diao K Xu H Liu X Predictors of aortic dilation in patients with coarctation of the aorta: evaluation with dual-source computed tomography. *BMC Cardiovasc Disord.* (2018) 18:124. 10.1186/s12872-018-0863-8 29929466 PMC6013956

[25] Sluysmans T Colan S. Theoretical and empirical derivation of cardiovascular allometric relationships in children. *J Appl Physiol.* (2005) 99:445–57. 10.1152/japplphysiol.01144.2004 15557009

[26] Meyer A Joharchi M Kundt G Schuff-Werner P Steinhoff G Kienast W. Predicting the risk of early atherosclerotic disease development in children after repair of aortic coarctation. *Eur Heart J.* (2005) 26:617–22. 10.1093/eurheartj/ehi037 15618050

[27] Rudolph A Heymann M Spitznas U. Hemodynamic considerations in the development of narrowing of the aorta. *Am J Cardiol.* (1972) 30:514–25. 10.1016/0002-9149(72)90042-2 4672503

[28] Vigneswaran T Sinha M Valverde I Simpson J Charakida M. Hypertension in coarctation of the aorta: challenges in diagnosis in children. *Pediatr Cardiol.* (2018) 39:1–10. 10.1007/s00246-017-1739-x 29043396

[29] Frandsen E Burchill L Khan A Broberg C. Ascending aortic size in aortic coarctation depends on aortic valve morphology: understanding the bicuspid valve phenotype. *Int J Cardiol.* (2018) 1:106–9. 10.1016/j.ijcard.2017.07.017 29169748

[30] Nadorlik H Bowman J Fitzgerald-Butt S Mah M McBride K Kovalchin J Abnormal longitudinal growth of the aorta in children with familial bicuspid aortic valve. *Pediatr Cardiol.* (2017) 38:1709–15. 10.1007/s00246-017-1740-4 28948327 PMC5798863

[31] Sehested J Baandrup U Mikkelsen E. Different reactivity and structure of the prestenotic and poststenotic aorta in human coarctation. Implications for baroreceptor function. *Circulation.* (1982) 65:1060–5. 10.1161/01.CIR.65.6.1060 7074769

[32] Keshavarz-Motamed Z Rikhtegar Nezami F Partida R Nakamura K Staziaki P Ben-Assa E Elimination of transcoarctation pressure gradients has no impact on left ventricular function or aortic shear stress after intervention in patients with mild coarctation. *JACC Cardiovasc Interv.* (2016) 9:1953–65. 10.1016/j.jcin.2016.06.054 27659574 PMC5402700

[33] Dharmashankar K Widlansky M. Vascular endothelial function and hypertension: insights and directions. *Curr Hypertens Rep.* (2010) 12:448–55. 10.1007/s11906-010-0150-2 20857237 PMC2982873

[34] Beaton A Nguyen T Lai W Chatterjee S Ramaswamy P Lytrivi I Relation of coarctation of the aorta to the occurrence of ascending aortic dilation in children and young adults with bicuspid aortic valves. *Am J Cardiol.* (2009) 103:266–70. 10.1016/j.amjcard.2008.09.062 19121449

[35] Sinning C Zengin E Kozlik-Feldmann R Blankenberg S Rickers C von Kodolitsch Y Bicuspid aortic valve and aortic coarctation in congenital heart disease-important aspects for treatment with focus on aortic vasculopathy. *Cardiovasc Diagn Ther.* (2018) 8:780–8. 10.21037/cdt.2018.09.20 30740325 PMC6331380

